# Recent understanding of the mechanisms of the biological activities of hesperidin and hesperetin and their therapeutic effects on diseases

**DOI:** 10.1016/j.heliyon.2024.e26862

**Published:** 2024-02-28

**Authors:** Zhongkai Ji, Wei Deng, Dong Chen, Zhidong Liu, Yucheng Shen, Jiuming Dai, Hai Zhou, Miao Zhang, Hucheng Xu, Bin Dai

**Affiliations:** Binhai County People's Hospital, No.148, Middle Fudong Road, Dongkan Town, Binhai County, Yancheng City, 224500, China

**Keywords:** Anti-inflammatory, Antioxidant, Antitumor, Antibacterial, Hesperidin, Hesperetin

## Abstract

Flavonoids are natural phytochemicals that have therapeutic effects and act in the prevention of several pathologies. These phytochemicals can be found in lemon, sweet orange, bitter orange, clementine. Hesperidin and hesperetin are citrus flavonoids from the flavanones subclass that have anti-inflammatory, antioxidant, antitumor and antibacterial potential. Preclinical studies and clinical trials demonstrated therapeutical effects of hesperidin and its aglycone hesperetin in various diseases, such as bone diseases, cardiovascular diseases, neurological diseases, respiratory diseases, digestive diseases, urinary tract diseases. This review provides a comprehensive overview of the biological activities of hesperidin and hesperetin, their therapeutic potential in various diseases and their associated molecular mechanisms. This article also discusses future considerations for the clinical applications of hesperidin and hesperetin.

## 1 Introduction

Citrus flavonoids, including hesperidin (Hsd) and hesperetin (Hst), have a wide range of biological effects. Citrus fruits such as lemon, sweet orange, bitter orange, clementine are rich in Hsd. Hesperidin is also found in mint, *Hypericum perforatum* (St. John's wort), *Salvia miltiorrhiza* (red sage) [1]. Hesperidin and hesperetin are found in the same plant material, Hsd is a metabolite of Hst. Hesperidin can be readily extracted from citrus processing residue [2,3]. However, the extraction process for Hst is complicated and increases the cost to end users.

**Fig. 1:**
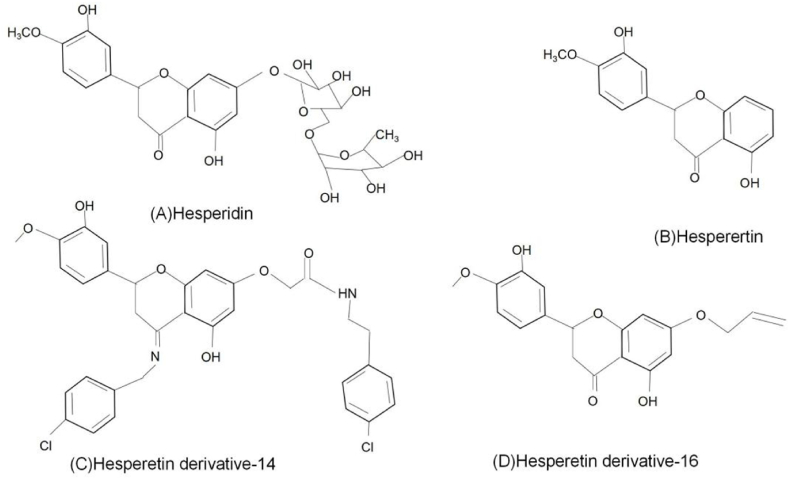
Chemicai structure of Hsd(A)、Hst(B)、HD-14(C) and HD-16 (D).

Hesperidin (C_28_H_34_O_15_), a flavanone glycoside, which is composed of aglycone Hst (C_16_H_14_O_6_) and a sugar moiety known as rutinoside. Rutinoside is a disaccharide composed of rhamnose and glucose, with glucose attached to C7 of the Hst ring. The chemical name for Hst is 4'-methoxy-3',5,7-trihydroxyflavanone (Fig. 1A), while that for Hsd is 4'-methoxy-3',5,7-trihydroxyflavanone-7-rhamnoglucoside (Fig. 1B) [4,5].

Hesperidin can be isolated using various methods such as extraction, percolation, batch or continuous reflux. The quality, yield, efficiency of extraction are influenced by different factors such as solvent type, temperature, extraction time and liquid-solid ratio. Common solvents in this extraction process include dimethyl sulfoxide (DMSO), methanol, or aqueous combinations of these solvents in various ratios. The another newer methods have replaced impregnation and Soxhlet extraction, resulting in improved efficiencies and selectivities. Hesperidin offers advantages such as enhanced safety, minimal cumulative side effects, low effective dose. Mice were administered Hsd at dosages ≤5 percent to validate its safety and even after long-term administration, Hsd caused no mutation, toxicity, or carcinogenesis [6]. Only 10 percent of individuals undergoing hesperidin therapy present with mild-to-moderate side effects [7]. The effects of Hsd and Hst have been investigated on exercise performance [[8], [9], [10]]. Oral administration of Hsd, Hst, or orange juice can aid muscle recovery and enhance performance in elite and recreational athletes by optimizing oxygen and nutrient supply to the muscles and improving anaerobic performance.

This review summarizes the mechanisms underlying the biological activities and therapeutic roles of hesperidin and hesperetin in various diseases as well as potential considerations for their future clinical applications.

## 2 Hesperidin and hesperetin bioavailability

Both Hsd and Hst exhibit approximately 20 percent bioavailability [[11], [12], [13], [14], [15], [16]]. Owing to its limited water solubility, only a small quantity of Hsd molecules are released into the aqueous environment of the gastrointestinal tract [16]. P-glycoprotein in the alimentary canal eliminates ingested chemicals from the extracellular space and inhibits their absorption [17]. Therefore, hesperidin may be released into the external environment following absorption by the intestinal epithelium. Numerous efforts have been made to improve the water solubility of Hsd.

Hesperetin derivative-14 (HD-14) (Fig. 1C) was synthesized using Mannich base synthesis and its molecular formula is C_34_H_32_Cl_2_N_2_O_6_. Its chemical name is (S)-5-hydroxy-2-(3-hydroxy-4-methoxyphenyl)-7-{[4-(trifluoromethyl) benzyl] oxy} chroman-4-one and it exhibits improved water solubility and bioavailability compared to its parent compound. Hesperetin derivative-14 also demonstrated anti-inflammatory properties and prevented liver fibrosis in mice [18]. Hesperetin derivative-16 (HD-16) was synthesized by combining Hsd with various brominated aromatic and alkyl groups (Fig. 1D); its molecular formula is C_19_H_19_O_6_. The chemical name of the compound is (S)-7-(allyloxy)-5-hydroxy-2-(3-hydroxy-4-methoxyphenyl) chroman-4-one and it exhibits improved water solubility and activity compared to Hsd [19]. Hesperetin derivative-16 ameliorates CCl_4_-induced liver fibrosis in mice [20].

Cyclodextrin glucosyltransferase was used to synthesize 8α-GH by combining glucose with Hsd. The latter displayed a 3.7-fold higher bioavailability and 10,000-fold increased water solubility compared with Hsd [21]. Dietary 8α-GH significantly reduces white adipose tissue weight in mice [22]. Methylation improves flavonoid bioavailability, metabolic stability, tissue distribution and biological activity [23]. Alkaline conditions promote the formation of hesperidin methyl chalcone (HMC), which exhibit increased metabolic resistance and transport capacity compared to their precursor compounds [12]. Hesperidin methyl chalcone has shown efficacy in treating chronic venous insufficiency [24,25], colitis, arthritis and UVB irradiation-induced effects [[26], [27], [28]]. Hesperidin methyl chalcone also ameliorates high-fat diet-induced lipid and sugar metabolism disorders and increases energy expenditure by promoting fat degradation [29]. Hesperidin -7-*O*-glucoside exhibited superior efficacy compared with its parent compound in restoring the hepatic antioxidant system and releasing cytokines [30]. Hesperidin exhibits limited targeting and oral absorption. A mannose-6-phosphate bovine serum albumin-labelled Hsd liposome carrier system targeting hepatic stellate cells (HSCs) was developed to prevent liver fibrosis in rats [31]. Garg [32] developed sulfonated hesperidin (*S*-Hsd) as an inhibitor of sexually transmitted infection (STI)-causing bacteria, specifically *Chlamydia trachomatis* and gonococci, without affecting normal vaginal flora such as *Lactobacillus gasseri*. It provides protection against certain STIs without causing adverse effects on sperm or vaginal tissues [32].

## 3 Biological activities of Hsd and Hst

**Fig. 2:**
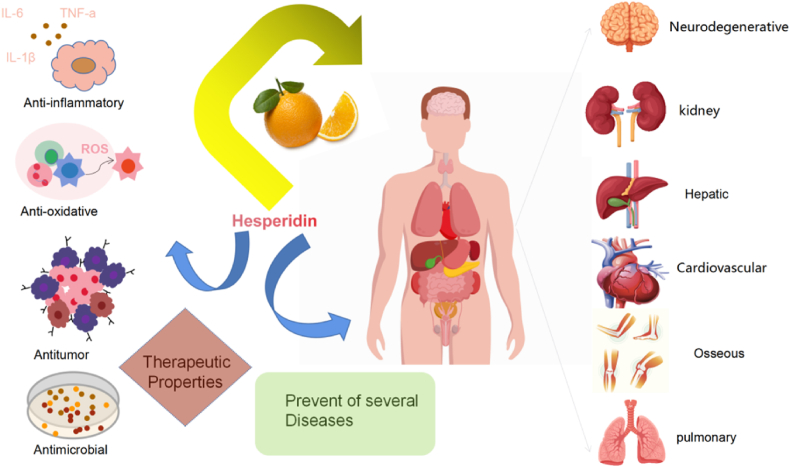
Biological activity of Hsd and Hst and its impact on diseases.

Hesperidin and hesperetin exhibit anti-inflammatory, antioxidant, antitumor and antimicrobial properties [33]. In recent years, various in vivo and in vitro studies have explored the therapeutic potential of Hsd and Hst in treating a range of diseases, including bone, cardiovascular, neurological, respiratory, digestive and urinary tract diseases (Fig. 2). Table 1 detailing the specific model, route, mechanism and reference of Hsd and Hst have been provided.

**Table 1:** In vitro and in vivo studies of Hsd and Hst for disease treatment.

Biological activities	Disease	Flavanone	In Vitro Models	In Vivo Models	Mechanism	Reference
Antioxidant	Arthritis	Hsd		Rheumatoid arthritis rats	Reduce the levels of catalase, nitric oxide and free radicals.	[140]
	Postmenopausal osteoporosis	Hst	RANKL-induced RAW 264.7 cells	Ovariectomized mice	Inhibit Jnk mediated Irf-3/c-Jun activation.	[141]
	Postmenopausal osteoporosis	Hst		Ovariectomized rats	Regulate bone morphogenetic protein pathway and osteopontin expression	[142]
	Genotoxicity	Hsd		Cyclophosphamide-induced mice	Reduce the frequency of MnPCE and increased proliferation on bone marrow cellularity that affected by CP.	[143]
	Cardiovascular Remodeling	Hsd		l-arginine methyl ester-induced rats	Down-regulation of TGF-β1 and TNF-R1 protein expression	[99]
	Metabolic Syndrome	Hsd	Bovine aortic endothelial cells		Stimulate phosphorylation of Src, Akt, AMP kinase, endothelial NO synthase to produce NO.	[144]
	Cardiomyocyte apoptosis	Hst	LPS-induced H9C2 cells		Downregulate the protein expression of Bax, upregulated the expression of Bcl-2 and attenuated the phosphorylation level of JNK	[145]
	Protect cardiomyocytes	Hsd		Senescent rats	Enhance the activity of enzyme antioxidants	[37]
	Myocardial ischemia reperfusion	Hsd		Experimentally-induced rats	Increase tissue nitrite, antioxidant level and Reduce inflammation, arrhythmias and apoptosis	[146]
	Metabolic syndrome and insulin resistance	Hsd		ISO-induced rats	Reduce levels of plasma cholesterol, LDL-C, VLDL-C, TG, FFA	[1]
	hypercholesterolemia and fatty liver	Hsd		High-cholesterol diet-induced rats	Inhibit synthesis and absorption of cholesterol and regulate the expression of mRNA for RBP, C-FABP, H-FABP	[147]
	Cognitive impairment	Hsd		APP/PS1 mice	Reduce the levels of ROS, LPO, protein carbonyl, 8-OHdG, increasing the activities of HO-1, SOD, catalase, GSH-Px	[148]
	Diabetes Depression	Hsd		Streptozotocin-induced diabetic rats	Enhancement of Glo-1 and activation of the Nrf2/ARE pathway	[149]
	Acute lung inflammation	Hsd		Ventilator-induced lung injury mice	Reduce recruitment of inflammatory cells to the airways and the formation of CCL-2 and IL-12	[150]
	Acute lung injury	Hst		LPS-induced ALI mice	(1)Inhibit MAPK activation, regulate Iκβ degradation, block the interaction MD2 and its co-receptor TLR4 (2)Associated with the TLR4-MyD88-NF-κβ pathway.	[151] [152] [153]
	Acute gastric injury	Hsd		Streptozotocin and nicotinamide-induced rats	Increase antioxidant defense capacity through the induction of HO-1 via ERK/Nrf2 pathway.	[154]
	Hepatotoxicity Nephrotoxicity	Hsd		Paclitaxel-induced rats	Amendment of Nrf2/HO-1 and caspase-3/Bax/Bcl-2 signaling pathways.	[155]
	Renal injury	Hsd		Aluminum-induced rats	Inhibit the MMP-9-related signaling pathway activated by ALCL3.	[138]
	Renal injury	Hsd		sodium fluoride-induced rats	Inhibit PI3K/Akt/mTOR pathway.	[156]
Anti-inflammatory	Postmenopausal osteoporosis	Hsd		Ovariectomized mice Ovariectomized rats	Inhibition of osteoclast superoxide reduces bone Resorption.	[157] [158] [159]
	Osteoporosis	Hst	RANKL-induced Murine macrophage cells	LPS-induced mice	(1)Inhibit activation of NF-κβ and MAPK signaling (2)Scavenger active oxygens (3)Activate the Nrf2/HO-1 signaling pathway.	[160]
	Cardiac hypertrophy	Hsd		Isoproterenol-induced rats	Interfering with NF‐κB signaling via JNK phosphorylation	[161]
	Hypertensive	Hsd		Spontaneously hypertensive rats	Improving NO bioavailability in endothelial cells.	[162]
	Hypertensive	Hsd	Human umbilical vein endothelial cells	Spontaneously hypertensive rats	Stimulate CaMKII/p38 MAPK/MasR expression and CaMKII/eNOS/NO production	[163]
	Parkinson's Disease	Hsd		6-OHDA-induced mice	Attenuate the striatal levels of proinflammatory cytokines tumor necrosis factor-α, interferongamma, interleukin-1β, interleukin-2, interleukin-6	[164]
	NeuroinflammationMemory Impairments	Hst	LPS-induced HT-22 cells BV2 cells	LPS-induced mice	Through the NF-κβ signaling pathway reduced the protein expression level of TNF-α and IL-1β cytokines	[165]
	Ischemic stroke	Hst		Middle cerebral artery occlusion mice	Inhibit the TLR4-NF-κβ pathway	[166]
	Temporal lobe epilepsy	Hst		Kainic acid-induced mice	Inhibit the expression of pro-inflammatory molecules	[167]
	Parkinson's Disease	Hsd		OHDA-induced zebrafish	Downregulated the kinases lrrk2 and gsk3β along with casp3, casp9, polg	[168]
	Asthma	Hsd		HDM-induced mice	Decrease subepithelial fibrosis, smooth muscle hypertrophy in airways, lung atelectasis to ameliorating airway structural remodeling	[169]
	Lung injury	Hsd	Acrolein-induced LLC cells	Acrolein-induced mice	Attenuate the expression levels of the activated forms of p38,p53, JNK	[170]
	Peptic ulcers	Hsd		Indomethacin-induced rats	Increase COX-2 and GSH expression	[171]
	Peptic ulcers	Hsd		Stress or ethanol-induced rats	Reduce neutrophil migration and strengthen the mucus barrier close to the mucosa	[172]
	Cholestasis	Hsd	FXR-suppressed HepaRG cells.	FXR-suppressed HepaRG mice	Activate farnesoid X receptor (FXR)	[173]
	Acute renal injury	Hsd		Cisplatin-induced rats	Attenuate caspase-3 and DNA damag.	[135]
	Hepatotoxicity	Hsd		Acetaminophen-induced rats	(1)Increase GSH, GST, SOD, GPx levels and deplete MDA level. (2)Elevate TNF-α and lower the levels of interleukin IL-4.	[174]
	Testicular and kidney damage	Hsd		Carbon tetrachloride-induced rats	Repaire renal and testicular architecture and suppress NF-κβ immunoexpression.	[175]
	Renal injury	Hsd		Colistin-induced rats	Decrease the levels of MDA and inflammatory parameters and Increase GSH, SOD, CAT, GSH-Px levels.	[136]
	Acute kidney injury	Hst		Cisplatin-induced rats	Activate Nrf2, and attenuate the MAPK signaling pathway.	[176]
Anticancer	Osteosarcoma	Hsd	MG-63 cells	BALB/c mice	Inhibition of cell migration and invasion, cell cycle arrest and induction of mitochondrial-mediated apoptosis.	[177]
	Glioma	Hsd		C6 glioma cells graft rats	Inhibit the proliferation of cerebrally implanted C6 glioma and involves suppression of HIF-1α/VEGF pathway	[178]
	Lung cancer	Hsd	NSCLC A549 cells		(1)Induce apoptosis through the mitochondrial apoptotic pathway and induced G0/G1 arrest. (2)inhibite the migratory and invasive capabilities of lung cancer cells by the mediation of the SDF-1/CXCR-4 signaling cascade.	[58] [179]
	Lung cancer	Hsd	Cisplatin-induced A549 cells		Decrease the expression of P-gp and increased the intracellular accumulation of the P-gp substrate, rhodamine 123	[180]
	Lung cancer	Hsd	LLC cells	Lung carcinoma mice	Increase pinX1 protein expression.	[181]
	Gastric cancer	Hsd		MNNG-induced rats	Activate autophagy and the PI3K/AKT pathway.	[182]
	Esophageal cancer	Hst	Eca109 cells	Human xenograft tumor nude mice	Depletion of GSH, accumulation of ROS, increase of cleaved caspase-9 and cleaved caspase-3	[183]
	Ehrlich ascites carcinoma	Hsd		Ehrlich ascites carcinoma (EAC) mice	Down-regulate Bcl2 and Stimulate Caspase3 and Bax genes expression	[184]
	Liver cancer	Hsd		DEN/CCl4-induced rats	Activate Nrf2/ARE/HO-1 and PPARγ pathways.	[185]
	Gastric cancer	Hsd		Xenograft tumor nude mice	Activate the mitochondrial pathway by increasing the ROS.	[129]
	Urinary-bladder carcinogenesis	Hsd		N-butyl-N-nitrosamine-induced mice	Decrease cell proliferation by the induction of cell differentiation.	[186]
Antimicrobial	Severe acute respiratory syndrome coronavirus 2 infection	Hsd	SARS-CoV-2 Spike Protein S1 Subunit-Induced A549 cells		Attenuated inflammasome machinery protein expressions (NLRP3, ASC, Caspase-1), as well as inactivated the Akt/MAPK/AP-1 pathway.	[187]
		Hst	ATCC CRL-1739 cells	ATCC 49503 ATCC 43504 ATCC 51932 ATCC700392	(1)block the expression of genes involved in H. pylori's transcriptional (rpoA, rpoB, rpoD, rpoN) and replication (dnaE, dnaN, dnaQ, holB) machinery. (2)reduce the expression of genes involved in H. pylori adhesion and motility (sabA, alpA, alpB, hpaA, hopZ)	[188]

### 3.1 Antioxidant capacity

Oxidative stress is a consequence of elevated levels of free radicals, specifically reactive oxygen species (ROS) or reactive nitrogen species (RNS), in response to various harmful stimuli. An imbalance between oxidants and antioxidants in the body can result in tissue damage. Reactive oxygen species encompasses the superoxide anion, hydrogen peroxide and hydroxyl radicals, whereas RNS encompasses nitric oxide (NO), nitrogen dioxide and peroxynitrite. These highly reactive molecules are directly or indirectly involved in maintaining the oxidative and antioxidative equilibrium within the body and interact with key signaling molecules to activate various pathways, including the nuclear factor kappa-light-chain-enhancer of activated B cell (NF-κB) signaling pathway, mitogen-activated protein kinase (MAPK) signaling pathway and mitochondrial apoptosis pathway.

**Fig. 3:**
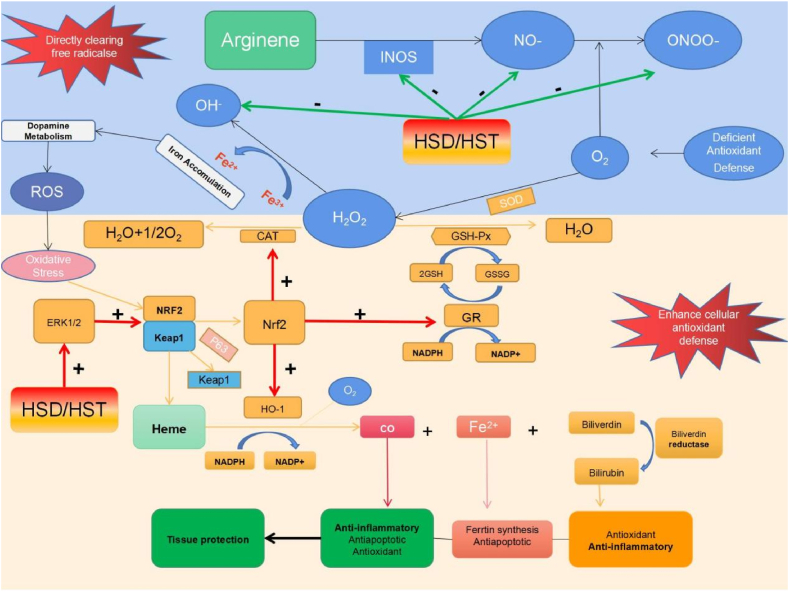
Basic cellular mechanism of antioxidant activity of Hsd and Hst.+, Raise/activate; -, Deactivate/suppress.

Hesperidin and hesperetin enhance cellular antioxidant defense by eliminating free radicals and ROS (Fig. 3). Specifically, hesperidin and hesperetin effectively neutralize ROS, including superoxide anions, hydroxyl radicals, peroxynitrite and NO radical [[34], [35], [36]]. The upregulation of nuclear factor erythroid 2-related factor 2 (Nrf2) and extracellular signal-related kinases (ERK) 1/2 further enhances cellular antioxidant defense [37,38] by increasing heme oxygenase (HO-1) expression, reducing intracellular pro-oxidants and increasing endogenous antioxidant bilirubin. Heme oxygenase then increases carbon monoxide (CO) levels in anti-apoptotic and anti-inflammatory cells and induces guanylate cyclase [39]. Nuclear factor erythroid 2-related factor 2 upregulates the expression of catalase (CAT), superoxide dismutase (SOD) and glutathione-*S*-transferase (GST). Additionally, the ERK/Nrf2 signaling pathway induces HO-1 expression, which further enhances the cellular antioxidant defense [39].

These studies indicate that Hsd and Hst possess antioxidant activity and potential efficacy.

### 3.2 Anti-inflammatory effects

Oxidative stress frequently elicits an accompanying inflammatory response through the activation of thioredoxin (Trx)-interacting protein (Txnip), which binds to the nucleotide-binding oligomerization structural domain-like receptor protein 3 (NLRP3). This interaction facilitates the binding of NLRP3 to apoptosis-associated speck-like protein containing a CARD domain (ASC) and cysteine aspartate protease-1 (Caspase-1), leading to activation of the NLRP3 inflammatory vesicle and increased secretion of the proinflammatory mediator interleukin 1β (IL-1β) [40,41]. Nuclear factor kappa-B transcription factors are activated, leading to transcriptional upregulation of inflammatory cytokine genes such as tumor necrosis factor alpha (TNF-α), IL-1β, IL-6, inducible nitric oxide synthase (iNOS) and cyclooxygenase 2 (COX-2) [42]. Nuclear factor kappa-B signaling pathway activation is closely associated with MAPK activation, which is a serine/threonine-specific protein kinase involved in intracellular signaling pathways during the proinflammatory response [43]. Additionally, intercellular adhesion molecule-1 (ICAM-1) and vascular cell adhesion molecule-1 (VCAM-1) can contribute to inflammatory signaling by regulating leukocyte-endothelial cell interactions [44,45].

**Fig. 4:**
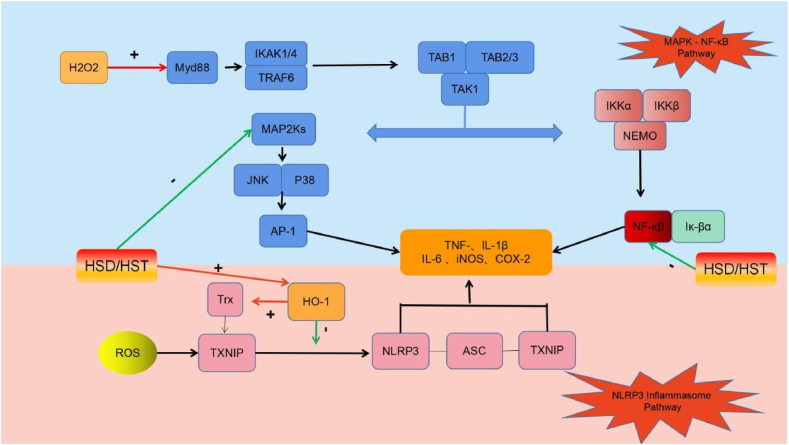
Basic cellular mechanism of anti-inflammatory activity of Hsd and Hst.+: Raise/Activate; -: Deactivate/suppress.

Hesperidin and hesperetin inhibit the Txnip/NLRP3, MAPK and NF-κB inflammatory pathways (Fig. 4). Additionally, hesperetin upregulates HO-1 expression, inhibits the activation of Txnip and its binding to NLRP3 and impedes the binding of NLRP3 to downstream caspase-1 and ASC, thereby inhibiting inflammatory body (inflammasome) activation and downregulating IL-1β levels [46]. Heme oxygenase also upregulates Trx expression, downregulates Txnip expression, promotes Txnip binding and indirectly inhibits inflammasome activation [47,48]. Hesperidin and hesperetin inhibit hydrogen peroxide-induced phosphorylation of ERK, c-Jun amino-terminal kinases (JNK), p38, NF-κB and NF-κB inhibitory protein (IκB) [49,50]. They also hinder IκB degradation, prevent NF-κB signaling pathway activation [51] and downregulate TNF-α and IL-1β as well as the inflammatory mediators IL-6, iNOS and CO. Furthermore, hesperidin suppresses MAPK kinase (MEK)/ERK phosphorylation in the MAPK signaling pathway, downregulates matrix metalloproteinase 9 (MMP-9) expression and reduces inflammation [52].

These findings highlight the anti-inflammatory properties and potential therapeutic efficacy of Hsd and Hst.

### 3.3 Anti-cancer efficacy

Cells undergo the cell cycle, a complex process facilitating growth and replication that is regulated by two main groups of genes: oncogenes (such as Ras and c-Myc) and tumor suppressor genes (such as p53) [53]. Cell cycle progression is regulated by the interplay between cyclin-dependent kinase (CDK) complexes, which consist of a CDK catalytic subunit and cyclin regulatory subunit. The primary CDKs involved in cell cycle progression are CDK1, -2, -4 and -6 [54]. Cyclin-dependent kinase complex activity is regulated by CDK inhibitors (CKIs), such as those from CIP/KIP (p21, p27, p57) and INK4 families [55]. The cell machinery orchestrates cell cycle progression by rhythmically modulating the synthesis and degradation of cyclins. The Tumor Suppressor Protein (P53) pathway, cellular energy levels and ratio of anti-apoptotic to pro-apoptotic proteins determine cell cycle arrest or apoptosis following DNA damage and/or mitochondrial dysfunction [56]. Tumor Suppressor Protein upregulates the expression of the CDK inhibitor p21 Cip1/Waf1 (P21), which forms complexes with CDK2, CDK-4, CDK-6 and suppresses the G1/S phase transition.

**Fig. 5:**
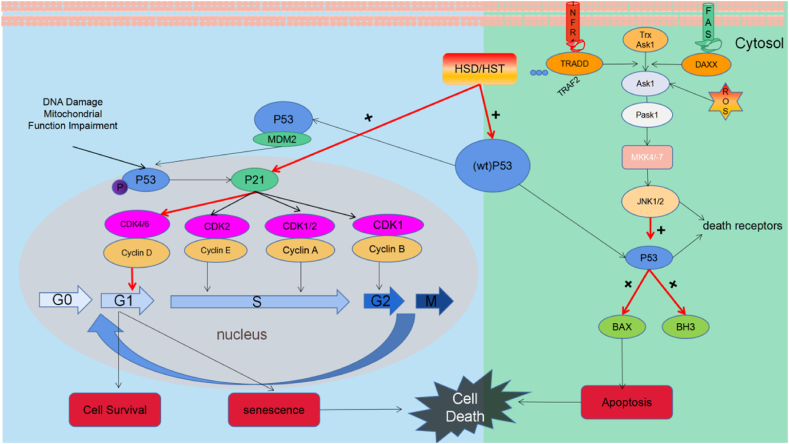
Basic cellular mechanism of anti-cancer activity of Hsd and Hst.+: Raise/Activate; -: Deactivate/suppress.

Hesperidin and hesperetin inhibit the G1/S transition in cancer cells through p53 (Fig. 5). Hesperidin increases the expression of wild-type p53 in breast cancer, lung cancer and leukemia cell lines in vitro and in colon cancer in vivo [[57], [58], [59]]. Hesperetin also upregulates wild-type p53 in SiHa cervical cancer cell line in vitro and in a breast cancer model in vivo [60,61]. The cyclin D-CDK4 complex phosphorylates the retinoblastoma protein pRb, leading to the generation of transcription factor (TF) E2F, which facilitates the G1/S transition and upregulates the expression of cyclin E and cyclin [62]. Additionally, circulation also upregulates cyclin E expression. Cyclin-dependent kinase 2 and cyclin E complexes phosphorylate downstream targets, thereby increasing DNA replication and facilitating progression to the S phase. Hesperidin upregulates p21 expression and downregulates cyclin D1 expression in A549 lung cancer cells, leading to G1 cell cycle arrest [58]. Hesperetin has similar effects on the ECA-109 esophageal cancer cell line [63]. It also downregulated cyclin E and CDK2 expression in HeLa cervical cancer and MCF-7 breast cancer cells. Therefore, it inhibits DNA replication [64,65]. C-Jun amino-terminal kinases-activated apoptosis is a crucial event in the MAPK pathway. C-Jun amino-terminal kinases influences cell death, proliferation and embryonic development. Increased FAS and TNF receptor activation, endoplasmic reticulum (ER) stress and cytosolic ROS levels lead to the dissociation and phosphorylation of apoptosis signal-regulating kinase 1 (ASK1) from Trx, resulting in the activation of JNK1/2 [56]. JNK1/2 and p53 regulate apoptosis. Hesperetin increased ROS levels in wild-type p53 MCF-7 breast cancer cells through phosphorylation of ASK1 and JNK [66]. It also upregulated JNK mRNA expression in the p53 mutant A431 cell line. Therefore, it may induce p53-independent apoptosis [67].

These findings highlight the anti-cancer activity and efficacy of Hsd and Hst.

### 3.4 Antimicrobial efficacy

Hyaluronidase performs multiple functions, including serving as a structural component in epithelial cell extracellular matrix (ECM) development, facilitating cell migration, participating in cell-cell signaling and triggering ECM remodeling enzyme activation and inflammation. Flavonoids exhibit antibacterial properties by forming complexes with hyaluronidase via electrostatic and hydrophobic interactions. Hesperidin and hesperetin inhibit bacterial DNA synthesis, reduce motility, decrease membrane permeability and downregulate metalloenzymes, while enhancing host defense [32,[68], [69], [70], [71], [72], [73], [74], [75]]. Hsd modifies the enzyme active site and reduces enzyme activity [76]. Both Hesperidin and hesperetin possess antifungal activity against *Botrytis cinerea*, *Trichoderma cinerea* and *Aspergillus fumigatus* [77,78].

Hesperidin has demonstrated antiviral efficacy against Severe Acute Respiratory Syndrome Coronavirus 2 (SARS-CoV-2) [79,80]. It inhibits the interaction between angiotensin-converting enzyme 2 (ACE2) and the receptor binding domain (RBD) of the SARS-CoV-2 spike protein [[81], [82], [83], [84]]. Hesperidin also exhibits strong binding affinity for other SARS-CoV-2 proteins, including 3-chymotrypsin-like protease (3CLPro/MPRO) and RNA-dependent RNA polymerase (RdRp) [85]. Hesperidin inhibits pp1a and pp1ab, the first proteins transported from the viral genome [84]. In contrast, hyaluronidase facilitates viral access to target cells and tissues, neutralizing the protective antiviral effect of Hsd in HeLa cells. Hesperidin exerts antiviral effects by inhibiting hyaluronidase [86]. The internal ribosome entry site is crucial for viral protein translation and is a promising therapeutic target for enterovirus 71 (EV-71). Hsd can potentially inhibit viral protein translation [87].

Hesperidin at 200 mg/ml concentration was highly effective in eliminating adult worms. However, at lower concentrations, the schistosomicidal effect was minimal or non-existent. These results suggest that hesperidin is effective only against adult worms at higher concentrations. The precise mechanism underlying the schistosomicidal effect of hesperidin in vitro remains unclear and warrants further investigation [88].

These findings indicate that Hsd exhibits antiviral, antibacterial, antifungal and antiparasitic activities. However, further research is required to elucidate the underlying mechanisms and to optimize the clinical dosage and form of administration for various applications.

## 4 Hesperidin and hesperetin as therapeutic agents

### 4.1 Bone diseases

Bone, a complex tissue composed of a mineralized organic matrix and various cells, is highly resistant to mechanical stress [89]. Healthy bone metabolism involves a delicate balance between bone generation and resorption [90]. However, aging and disease can disrupt this process and affect bone metabolism [91]. Multiple studies have demonstrated that increased levels of proinflammatory cytokines, including TNF-α and IL-1β, significantly contribute to the pathogenesis of bone diseases [92]. In particular, TNF-α downregulates the synthesis of major ECM components and disrupts cartilage by inhibiting the anabolic activities of chondrocytes [93,94]. Additionally, TNF-α stimulates the production of MMPs, particularly MMP-13, by activated chondrocytes, further contributing to cartilage degradation [91]. In addition, the NF-κB signaling pathway, activated by TNF-α, facilitates the proinflammatory function of TNF-α [95]. In osteoarthritis (OA), inflammatory cytokines not only have destructive effects but also contribute to chondrocyte apoptosis and cartilage degeneration [92].

Hesperidin reversed TNF-α-induced upregulation of IL-1β, PTGS2 and MMP-13. Furthermore, Hesperidin inhibited TNF-α-induced degradation of the chondrocyte extracellular matrix and reversed TNF-α-induced inhibitory effects on chondrocyte proliferation [96]. Hesperidin reduces serum levels of RANKL, TNF, IL-1β and IL-6 receptor activators and increases osteocalcin levels in a mouse model of lipopolysaccharide (LPS)-induced osteoporosis [96].

Hesperidin and hesperetin possess anti-inflammatory and antioxidant properties that support bone cell metabolism, making them potential candidates for prevention and treatment of OA and osteoporosis.

### 4.2 Cardiovascular diseases (CVD)

Following myocardial infarction (MI), collagen-producing myofibroblasts are activated, contributing to the gradual development of replacement fibrosis. Myocardial fibrosis undoubtedly affects left ventricular remodeling. Myocardial fibroblast activation is triggered by myocardial fiber stretching and inflammation. Myocardial fibrosis is regulated by inflammation, various cell types, paracrine mechanisms (such as TGFs) and collagen-degrading enzymes (such as MMPs) [97].

Hesperidin inhibits both caspase-3 and myeloperoxidase and smooth muscle actin alpha (α-SMA) and MMP-2 play crucial roles in preventing cardiac dysfunction and myocardial remodeling following MI by inhibiting collagen deposition and fibroblast migration [98]. Hesperidin and hesperetin effectively prevented hypertension and cardiac remodeling in a rat model of N(ω)-nitro-L-arginine methyl ester (L-NAME)-induced hypertension, as evidenced by the reduction in left ventricular wall thickness, cross-sectional area (CSA), fibrosis, vascular remodeling and expression of transforming growth factor β1 (TGF-β1) and TNF-1 proteins [99].

Hesperidin upregulates serum and hepatic SOD and GSH-Px in low-density lipoprotein (LDL) receptor-deficient (LDLR^−/−^) mice fed a high-fat diet, which suggests an increased endogenous defense against oxidative stress [100]. Furthermore, hesperidin-treated animals exhibited less severe atherosclerosis than their control counterparts, as hesperidin-treated animals had lower serum oxidized (OX)-LDL, IL-6 and TNF levels than controls [100].

Therefore, hesperidin and hesperetin safeguards cardiovascular health by exhibiting anti-inflammatory and antioxidant properties, as well as other pharmacological effects.

### 4.3 Neurological diseases

Alzheimer's disease (AD) is caused by the accumulation of amyloid beta (Aβ) plaques and various mechanisms, such as the generation of ROS [101] and the activation of astrocytes and microglia [102]. Nuclear factor erythroid 2-related factor 2 regulates endogenous antioxidant mechanisms [103,104]. Elevated ROS levels lead to the downregulation of Nrf2 and HO-1 (heme oxygenase-1), contributing to the pathogenesis of AD-like effects [105]. Microglia are tissue macrophages that perform tissue maintenance and immune surveillance [106]. Activated microglia express various pattern recognition receptors, specifically from the Toll-like receptor (TLR) family, which enable them to detect microbial invaders. Toll-like receptor activation exacerbates various signaling pathways, resulting in the activation of inflammatory agents such as cytokines, ROS and NO [107]. Microglial expression of TLRs in the central nervous system (CNS) is the first line of defense against endogenous and exogenous agents [107,108]. Toll-like receptor participate in Aβ signaling, triggering an intracellular mechanism that leads to the activation of proinflammatory agents and clearance of Aβ [[109], [110], [111]]. Toll-like receptor-mediated inflammatory responses induced by Aβ can result in neurotoxicity. Toll-like receptors 2, 4 and 9 have been suggested as potential therapeutic targets for AD treatment [112,113].

Hesperidin mitigates LPS-induced neuroinflammation, cell death and memory impairment via TLR4/NF-κB signal transduction. Hesperidin improves TLR4-mediated ionized calcium-binding adapter molecule 1/glial fibrillary acidic protein (Iba-1/GFAP) and downregulates inflammatory cytokines, leading to a decrease in LPS-induced memory impairment. Hesperetin efficiently reverses the pathological outcomes of Aβ treatment in mice and cells, primarily by inhibiting oxidative stress via regulation of Nrf2/HO-1, reducing neuroinflammation by regulating TLR4/NF-κB and preventing apoptotic cell death by regulating Bax/Bcl-2, Caspase-3 and Poly (ADP-ribose) polymerase 1 (PARP-1) in the Aβ mouse model and in cells [114].

Hesperidin and hesperetin demonstrate potential as novel therapeutic agents for the treatment of AD-like neurodegenerative disorders owing to their anti-inflammatory and antioxidant properties.

### 4.4 Respiratory diseases

Idiopathic Pulmonary fibrosis (IPF) is primarily characterized by injury to the epithelial cells (ECs), increased levels of alpha smooth muscle actin (α-SMA, one of the myofibroblast markers) and ECM in the alveolar walls, myofibroblast accumulation and matrix remodeling resulting in distorted alveolar structure and lung parenchyma remodeling. This leads to progressive alterations in lung functions [115], including pulmonary edema, inflammation and fibrosis. Elevated levels of ROS due to pulmonary insults have been linked to the release of proinflammatory cytokines such as TNF-α, IL-1β and IL-6 during the initial stages [116]. Transforming growth factor β1 has been implicated in the differentiation of myofibroblasts from fibroblasts, excessive formation of myofibroblasts results in the elevated synthesis and deposition of ECM proteins in the lungs [117]. Transforming growth factor β has also been reported to stimulate ROS generation via Smad 2/3 and MAPK signaling activation [118]. Adenosine monophosphate-activated protein kinase (AMPK) is a key element in pulmonary fibrosis and its activation results in autophagy and decreased levels of collagen and ECM. Inhibition of AMPK activation by TGF-β results in mitochondrial dysfunction, which upregulates mitochondrial ROS formation to induce epithelial cell injury [119].

Hesperidin downregulates the TGF-β1/Smad3/AMPK and NF-κB pathways, resulting in improved regulation of oxido-inflammatory markers (such as Nrf2 and HO-1) and proinflammatory markers (such as TNF-α, IL-1β, IL-6) and reduced collagen deposition during pulmonary fibrosis [120]. In a mouse model of bleomycin-induced pulmonary fibrosis, hesperidin downregulated the IL-6/signal transducer and activator of transcription 3 (STAT3) pathway, resulting in upregulation of P53, p21 and p16 (myofibroblast markers). Additionally, hesperidin downregulated α-SMA, reduced the number of aging-related β-galactosidase-positive cells, prevented lung fibroblast aging and ameliorated pulmonary fibrosis [121].

The anti-inflammatory, antioxidant and anti-cancer properties of Hsd and Hst suggest their potential clinical application in the treatment of respiratory diseases, including pulmonary fibrosis.

### 4.5 Digestive diseases

Hesperidin has numerous health benefits, including protection against digestive system diseases. Extracellular matrix can accumulate and form fibrotic tissue in response to chronic liver injury, leading to cirrhosis and liver failure in severe cases. Activated hepatic stellate cells (AHSC), a subset of α-SMA-positive liver fibroblasts, contribute to ECM formation during fibrosis. The activation of AHSCs is triggered by liver inflammation, which in turn leads to fibrosis [122,123]. Chronic inflammation and extracellular matrix accumulation cause liver parenchyma fibrosis and ultimately result in scar tissue formation [124,125]. Liver macrophages release proinflammatory cytokines and fibrotic mediators that activate dormant HSCs and transform them into myofibroblasts [126,127].

Hesperidin has demonstrated antifibrotic effects in various models of liver fibrosis, including a rat model of CCl_4_-induced liver fibrosis and primary HSCs. In a mouse hepatic fibrosis model, hesperidin upregulated tissue inhibitor of metalloproteinases 1 (TIMP-1) and suppressed the primary hepatic macrophage inflammatory response. The antifibrotic action of hesperidin is associated with Hedgehog signaling [18] and it protects human hepatocytes against *tert*-butyl hydroperoxide (tBuOOH)-induced oxidative damage [128]. Hesperidin also mitigated hydrogen peroxide (H_2_O_2_)-induced hepatic L02 cell injury by upregulating HO-1 [39].

Additionally, hesperidin upregulates the ROS-activated mitochondrial pathway, thereby inhibiting proliferation and promoting apoptosis of stomach cancer cells [129]. Moreover, hesperidin promotes apoptosis in the HT-29 colon cancer cell line via a Bax-dependent mitochondrial mechanism involving oxidant/antioxidant dysregulation [130].

### 4.6 Urinary tract diseases

Emerging evidence suggests that proinflammatory cytokine release (specifically TNF-α) [131], infiltration of inflammatory cells, such as macrophages and leukocytes [132] and mitochondrial dysfunction [133] are implicated in the pathogenesis of acute kidney injury. Additionally, nephrotoxic drugs induce renal tissue necrosis and apoptosis by activating caspase-3, a key player in the execution phase of apoptosis [134].

Hesperidin has demonstrated a range of potential therapeutic effects in acute renal injury, including reducing oxidative stress, inflammation and DNA damage [135]. In addition, hesperidin and chrysin attenuated myxin-induced nephrotoxicity [136]. Hesperidin has also been shown to protect rats against acrylamide-induced nephrotoxicity, oxidative stress, lipid peroxidation and DNA damage [137]. Hesperidin modulates MMP-9 expression and apoptosis and protects against Al-induced kidney damage in rats [138] and acute As-induced liver and kidney injury in mice [139]. Transforming growth factor β1 upregulation in hypertensive rats is associated with endothelial dysfunction and remodeling. Transforming growth factor β-stimulated pericyte myofibroblast differentiation and proliferation induces kidney damage and fibrosis. Hesperidin significantly reduces serum ACE and plasma TGF-β1 activity and downregulates renal angiotensin II receptor type 1 (AT1) [138]. Thus, hesperidin prevents L-NAME-induced hypertension, vascular and renal dysfunction, renal artery remodeling, glomerular ECM accumulation and renal fibrosis.

## 5 Conclusion

Oxidative stress and inflammatory response are interconnected and interact with each other. Oxidative stress triggers inflammation, which in turn increases the production of ROS, thereby exacerbating oxidative damage. Hesperidin and hesperetin enhance cellular antioxidant defense by eliminating free radicals and ROS. Additionally, Hesperidin and hesperetin inhibit the Txnip/NLRP3, MAPK and NF-κB inflammatory pathways, their upregulates HO-1 expression, inhibits the activation of Txnip and its binding to NLRP3 and impedes the binding of NLRP3 to downstream caspase-1 and ASC, thereby inhibiting inflammatory body activation and downregulating IL-1β levels. The Tumor Suppressor Protein (P53) pathway, cellular energy levels and ratio of anti-apoptotic to pro-apoptotic proteins determine cell cycle arrest or apoptosis following DNA damage and/or mitochondrial dysfunction. Hesperidin and hesperetin inhibit the G1/S transition in cancer cells through p53. Recent studies have shown that hesperidin and hesperetin exhibits antiviral, antibacterial, antifungal and antiparasitic activities. Although their exact mechanism of action is not fully understood, several mechanisms have been proposed, such as activation of the host immune system, disruption of bacterial membranes and interference with microbial enzymes.

Hesperidin and hesperetin are of natural origin, low toxicity, easy to obtain, and have good prospects for development and utilization in the future. However, due to the poor solubility of Hsd and its susceptibility to hydrolysis by gastric acid and enzymes, the bioavailability is low. Hesperetin is more stable than Hesperidin and has a higher bioavailability, but it is easily metabolized in the body, making it difficult to maintain a higher blood concentration, therefore, it is a major focus to carry out a certain amount of chemical modification of Hsd and Hst to find a synthetic product with a good pharmacological efficacy and a high degree of bioavailability. In addition, in clinical studies, factors affecting the efficacy of flavonoids include the dose of flavonoid metabolites used, the patient population, and the duration of the study. In previous studies, we can note the lack of sufficient clinical information on the therapeutic effects of Hsd and Hst. Therefore, further clinical studies exploring the appropriate dose, bioavailability, efficacy and safety of Hsd and its metabolites are still needed.

## Data availability statement

No data was used for the research described in the article.

## Funding

This work was supported by the study on Hesperidin on Fibrosis of Frozen Shoulder Inflammation and Mechanism Research (KD2023KYJJ033) by the Kanda College of Nanjing Medical University Research and Development Fund for Natural Science General Programs.

## CRediT authorship contribution statement

**Zhongkai Ji:** Writing – review & editing, Writing – original draft, Software, Investigation, Funding acquisition, Formal analysis, Data curation. **Wei Deng:** Data curation. **Dong Chen:** Data curation, Conceptualization. **Zhidong Liu:** Data curation. **Yucheng Shen:** Conceptualization. **Jiuming Dai:** Data curation. **Hai Zhou:** Data curation. **Miao Zhang:** Data curation. **Hucheng Xu:** Data curation. **Bin Dai:** Formal analysis, Conceptualization.

## Declaration of competing interest

The author(s) declare financial support was received for the research, authorship, and/or publication of this article.
